# A Treatise on Mental Derangement

**Published:** 1825-04-01

**Authors:** 


*A Treatise on Mental Derangement, containing the substance of the Gulstonian Lectures, for May*, 1822. By FRANCIS WILLLIS, M.D. Fellow of the Royal College of Physicians. Octavo, pp. 234, London, 1823.

WE conceive that Dr. Willis was hardly prudent in styling this work a *Treatise* on mental derangement. The term *Essay* would have been more appropriate, and more in consonance with the substance of a Gulstonian lecture. The work is divided into seven chapters, in the first of which our author seems to have set up a man of straw for a serious combatant. Who, in the present state of our knowledge, denies the connexion between mind and body, and their mutual influences on each other? None, we believe. But, says our author, the disease under consideration has been looked upon by some as merely and exclusively a *mental* malady, curable, without the aid of medicine, by what are termed moral remedies—such as travelling, and various kinds of amusement. And so we believe it often is; for granting that it is a corporeal disease—that is, a disordered function of the brain, as the material organ of thought, may not moral remedies act, and very powerfully too, on this same organ of thought? If moral emotions can produce insanity, may not contrary emotions tend to cure it? This indeed is admitted and pointed out by Dr. Willis himself. We agree with him, that it is to the nervous system we are to look for the seat of insanity. Any deviation from a state of health in the nerves must occasion a derangement in the transmission of sensations to the sensorium, and, consequently, inaccuracy of perception there.

"My endeavour in the preceding observations is not to point out what constitutes a MIND, for that is wholly out of our reach; but to shew that the nerves are the medium of communication between the mind and the body, that the mind varies from alterations taking place in them, that mental derangement should be considered for the most part as primarily a disease of the body, particularly of the nerves; and that the cure ought to be attempted by means principally acting upon the body." 36.

It is quite unnecessary to follow our author's arguments in support of the above positions, which have long been acknowledged and acted upon by all intelligent physicians in this country and on the continent.

The second chapter of the work is entitled "*a Description*

*of the* High State;" which certainly is not a very scientific, though it may be an expressive term to apply to a particular condition of mental derangement.

"There are two states then of this malady, both of which may, in their progress, pass into delirium, and again subside into derangement, and both, by neglect and improper treatment, may end in insanity;—so that derangement, delirium, and insanity are to be regarded as different degrees of mental disorder." We confess that this passage does not brighten our ideas on the subject of insanity. Perhaps, however, it is proper that the language should be suited to the subject.

"One state of derangement is characterized by an unrestrained behaviour, by an irritability which urges on the patient in pursuit of something real or imaginary to the ruin of himself, and the annoyance of his friends, and ultimately leads him, if opposed in his disordered wishes, to acts of extreme violence.

"The other state is marked by an unusual lowness, sometimes amounting to despair, a loathing of life, and every thing connected with it, accompanied too often by an uncontrollable effort to rescue himself, by his own hand, from his real or imaginary distresses." 41.

These distinctions, in general, are sufficiently discernible; but each case varies with the constitution—some partaking so much of both states, being neither high nor low, that a person unused to see such might be easily deceived, and even think the patients well. In the high state, our author thinks, the whole nervous system is the seat of the disorder—in the low state, "the stomach and other viscera seem to be more especially affected." The distinction between delirium, mental derangement, and insanity, has been so ably drawn (our author thinks) by his late relative (Dr. R. D. Willis) in his examination before the House of Commons, in the year 1810, that he cannot improve upon it. It runs thus:—

'In *delirium*, the mind is actively employed upon past impressions, 'upon objects and former scenes, which rapidly pass in succession before 'the mind, resembling in that case, a person talking in his sleep; 'there is also a considerable disturbance in the general constitution, 'great restlessness, great want of sleep, and a total unconsciousness of 'surrounding objects. In *insanity*, there may be little or no disturbance 'apparently in the general constitution, the mind is occupied 'upon some fixed assumed idea, to the truth of which it will pertinaciously 'adhere, in opposition to the plainest evidence of its falsity; 'and the individual is always acting upon that false impression. In 'insanity, also, the mind is awake to objects which are present. Taking 'insanity, therefore, and delirium, as two points, I would place *derangement of mind* somewhere between them.' 44.

In the above passage we can perceive a distinction between delirium and insanity, as two opposite points; but we affirm that there is no distinction laid down between either of them, and that "no man's land," which the doctor has placed "somewhere between them." Neither does the younger Willis at all, in our apprehension, elucidate the definition of his relative, by his own commentary.

"It will be evident from this definition, that delirium is distinct from insanity, neither can there be any difficulty in distinguishing delirium from mental derangement; because an unconsciousness of surrounding objects, together with much mental and *bodily disturbance*, are the characteristic symptoms of the former. But the greatest caution is sometimes necessary in distinguishing mental derangement from insanity. The *characteristic* symptoms of insanity are a firm *belief in an assumed idea*, upon which the patient is always acting, without any apparent bodily disease. In mental derangement there is also *a firm belief in an assumed idea*, upon which the patient is continually acting, but with this difference, that it is always accompanied with bodily disease. This amounts, sometimes, almost to as much as attends delirium; at other times it is apparently so trifling as scarcely to be discovered, even by those who are conversant with the disorder." 45.

If the author had any clear ideas of his own in writing the above passage, he has failed in communicating them to us. This, however, may be from want of proper apprehension on our part. Our author next proceeds to describe the "advancement and progress" (which, by the bye, must be pretty nearly synonimous in this case) of the high state of mental derangement. This description he prefers giving from Dr. Monro rather than from his own observations.

"'High Spirits, as they are generally termed, are the first symptoms 'of this kind of disorder; these excite a man to take a larger quantity 'of wine than usual; for those who have fallen under my observation 'in this particular, have naturally been very sober, and the person thus 'afflicted, from being abstemious, reserved, and modest, shall become 'quite the contrary, drink freely, talk wildly, obscenely, swear, sit up 'till midnight, sleep little, rise suddenly from bed, go out a hunting, 'return again immediately, set all his servants to work, and employ 'five times the number that is necessary; in short, every thing he does 'or says, betrays the most violent agitation of mind, which it is not in 'his own power to correct; and yet, in the midst of all this hurry, he 'will not misplace one word, or give the least reason for any one to 'suppose he imagines things to exist, that really do not, or that they 'appear to him different from what they do to other people. They 'who see him but seldom, admire his vivacity, are pleased with his 'sallies of wit, and the sagacity of his remarks, nay, his own family are

'with difficulty persuaded to take proper care of him, until it becomes 'absolutely necessary from the apparent ruin of his health and for-'tune.'" 48.

It is evident that the above picture was copied from the acts of a man of property, whose establishment was large, and who was fond of field sports. Each individual presents peculiarities according to his habits and employments prior to the mental aberration. The literary man would be occupied with his books, the tradesman with his accounts, the soldier with his arms and evolutions, &c. All their actions and expressions, however, would be characterised by an unusual hurry and bustle, indicative of the excitement going on in the sensorium.

These symptoms are only prelusive of others much worse. Conversation becomes irksome—relations appear unkind in their behaviour—those most loved become objects of aversion—in short, the unfortunate man takes up a firm belief in an assumed or visionary idea, which may frequently be traced to something that had engaged his attention in the early period of his illness.

"To constitute derangement of mind, his aberrations must be attended with bodily indisposition. If the latter be not apparent, the chance of cure is hereby diminished; the case then partaking more of insanity than derangement. If, on the other hand, the symptoms of bodily indisposition increase, delirium ensues; and then the patient 'begins to rave, and talk wildly and incoherently; swears, as if in the 'most violent rage; and then immediately after, bursts out into fits of 'laughter; talks obscenely, directs offensive and contemptuous language 'against his relations, and those around him; spits at them; destroys 'every thing that comes in his way; emits loud and discordant screams, 'and continues in this way till he is quite exhausted. The state of rest 'which follows, is generally short and sleepless; the patient is obstinate, 'will not speak one word; clenches his teeth if any thing is offered him 'to swallow; or else, with a degree of cunning, he pretends to drink a 'little; but immediately squirts it out again on the person who offered 'it. At once, however, he again breaks out into all the wild and extra-'vagant language and actions he committed before. If kept in strict 'coercion, he has often so much command over himself as to behave 'mildly and modestly; and were it not for the general expression of 'his countenance, and the peculiar glistening appearance and rapid move-'ment of his eyes, he might impose on many of the bye-standers, and 'make them imagine that the state of phrenzy was over."*

These symptoms vary much, according to the idiosyncrasy of the patient. In some, the irritation never rises to delirium.

---

* Vide vol I. p. 160. An Enquiry into the Nature and Origin of Mental Derangement, by Alexander Crichton, M.D.

When it does, the length of the paroxysm is various—from a few minutes to 24 or 48 hours, during which the patient will talk incessantly, till apparently worn out and exhausted. After a short repose, he will begin again, more violently than ever—especially if sleep should have recruited the body.

Such are the *mental* phenomena. The *corporeal* are next to be examined. Irritability and constant motion of the body, are the first singularities that strike the observer. As the irritability increases, the patient becomes unruly, and mischievous—breaking or destroying every thing that comes in his way. The countenance appears mottled—the eyes exhibit a singular wildness, often amounting to the terrific. The pulse varies, but is generally quick—the tongue is white and tremulous. Thirst is not usually complained of, but salivation not unfrequently takes place. The temperature also varies, but the head is often very hot, whilst the feet, hands, and fingers are cold as marble. The patient appears to experience great distress in the præcordia—the respiration is hurried—the breath hot and offensive. He will eat voraciously, and indiscriminately. The internal senses are affected, as well as the sensorial functions. The stomach will not act by emetics, nor the bowels by purgatives. A blister produces little pain—and heat or cold is not much attended to by the patient. "In short, as Dr. Willis properly observes, every organ will be found more or less disordered, and the natural sense throughout the frame perverted." 60. No one will deny that here there is evidence of corporeal disease. In short, no man who is not insane himself, will argue for *purely mental* disease. In every case of insanity, the organ, through which the mind is made manifest, is deranged in function—and it is in this way only, that the phenomena of insanity become known to us.

The 3rd chapter of the work before us, is on the remote and proximate causes of insanity. Dr. Willis very properly objects to Dr. O'Halloran's distinction between mania from a moral, and mania from a physical cause. The two may be identical as to pathology. Moral causes, as we said before, may produce lesions of function or structure in the brain, and thus lead to insanity. The same lesions of function or structure may be the result of physical causes—as inebriety, for example—and still the mental aberration will be the same. We agree, therefore, with Dr. Willis in the following passage.

"I cannot but differ widely from Dr. Halloran in his practical distinction; for, whether the disorder is produced by causes that immediately affect the mind, as disappointed ambition, and loss of fortune, or those that act primarily on the body, (such as excess in drinking, and other sensual desires) the disorder, when produced, is identically the same, owing its origin to disease of the body, which is to be cured by means more immediately applied to, and acting upon, the body. It does not follow that every man who loses his fortune, or meets with disappointment, is to become deranged; but, if either of these circumstances operate so powerfully upon the mind as to cause sleepless nights, for any length of time together, the body necessarily suffers for want of its natural rest, and the health is hereby as much disordered, as it would be after spending many nights in continued intoxication. In both these examples, the sufferers are rendered incapable of attending to business; they alike become confused, restless, and irritable, and liable to be deranged, which refreshing sleep, in both cases, at an early period, might prevent. An attempt to cure such, however, by remedies which act solely on the mind, would be altogether vain. Were we to shut a deranged man up in a room, allow him no exercise, give him no medicine, and thus make no use of bodily applications, but of arguments and mere mental amusements, we should clearly leave the more effectual remedies untried." 66.

Dr. Willis has somewhat unguardedly, we think, filled several pages with quotations and illustrations from Shakspeare's King Lear, to shew that, although the mind may be primarily affected, the body suffers previous to any mental derangement. The poet's illustrations are all very natural and correct, but they are not calculated for a pathological discussion, as Dr. Willis, we are sure, will allow, on sober reflection.

In respect to the proximate cause, or exact condition of the body, during insanity, there is great difference of opinion. Dr. Cullen supposes it to depend upon a peculiar state of the nervous system—Crichton, on a specific diseased action of those fine vessels that secrete the nervous fluid of the brain—Hill, to the same effect—Cox, Arnold, Mayo, place insanity to the account of a determination of blood to the brain, or an increased activity in its vessels—while Esquirol avers. that it is not necessarily connected with any one particular disease of structure—but that merely the function of the sensorium is disordered, and that from a variety of causes, moral and physical.

Our author considers the structural lesions, discoverable on dissection, as the consequences rather than the causes of insanity—and in this he is supported by most of the best pathologists of the present day. He considers Cullen's opinion (above quoted) as coming as near the truth as that of any of the modern writers on the disease.

Dr. Willis next examines the principal remedies that have been recommended in insanity, beginning with blood-letting. Dr. W. is a great enemy to this measure, and quotes, in his support, much Greek from the ancients, and, as usual, some poetry from Shakspeare. But what is rather more to the purpose, he refers to the success of his relative (Dr. Willis) as a proof that venesection is rarely necessary or proper, even in the high state of insanity described above.

"Although we suppose the brain to be more especially concerned in all the mental operations, it is to the region of the diaphragm that we still refer all painful and pleasurable sensations of the mind; it is in this region that the agony of grief, the pangs of disappointment, and the excess of joy, are so sensibly felt. Nothing, indeed, can operate strongly on the mind without in some degree disturbing the regularity of the functions of the viscera in this region; hence, in palpitation, syncope, sighing, sobbing, and convulsive laughter, which are all natural consequences of mental emotion, the heart, lungs, and diaphragm are more or less concerned.

"This did not escape Shakspeare, in his beautiful delineation of Lear's disorder; who, previous to his derangement, thus complains;—

"*Lear.* Oh, how this mother swells up toward my heart!
'*Hysterica passio!*——
'Oh me! my heart! my rising heart!"

"Whether, then, we look upon the disordered functions of the thoracic viscera as symptoms of bodily disease, or consider the extraordinary quickness of the imagination, the wild association of ideas, and the unnatural volubility of language, which occur in this complaint, as marks of mental disturbance, what term, I would ask, could be found more appropriate to designate this disease, than the one adopted by the ancients (phrenitis) which comprises the acute disorder both of the body and mind?

"Dissection has certainly disclosed that turgescence of the vessels of the brain, and thickening of its membranes, take place in this complaint; hence, the effect has been confounded with the cause, and hence, phrenitis and inflammation of the brain have become synonymous terms; but would it not be very remarkable, if the brain, the most delicate and essential organ in the body, exhibited no marks of disease, after the constitution had suffered such agitation and disturbance as I have heretofore described." 113.

In respect to the treatment, then, of the high state before described, our author remarks that, in general, the more violent the delirium, the more is death to be apprehended from exhaustion. We see, says he, the irritability of a man in his sound senses allayed by means which tend to procure rest, and give support to the system. "How much more necessary is it, then, that these same means should be prescribed when that irritability is greatly increased, and liable every moment to destroy life." We will allow our author to give a practical illustration of his doctrine.

"The patient was a young lady, of a naturally irritable constitution; who, having been in a very nervous state for many months, was, from domestic occurrences, thrown into a most violent delirium; on the sixth day from the attack of which she was placed under my care. With short intervals of cessation, she had been continually raving for four successive days and nights; labouring, at the same time, under such irritability, that four persons had been employed to watch and prevent her from getting out of her bed. While in this state, and previously to her becoming my patient, leeches had been applied to her forehead and temples, cupping-glasses to the back of her neck, and a blister to her head; purgatives, also, were given; barley-water with weak broth had been the only sustenance allowed. Her state, as I found it, was this: she had ceased to rave, probably from exhaustion, having been wholly without sleep; she had become obstinately silent, but was still in perpetual motion; her pulse was 130, her whole skin very hot, and completely parched; her face flushed and bloated; her eyes suffused with blood, and wide open; yet she could discern nothing; she was also unconscious of her evacuations; her tongue was brown, her lips and teeth covered with sordes. In attempting to feed her with a spoon, she clenched her teeth; if we succeeded in putting any thing into her mouth, she spit it out, after keeping it there a moment; so that it was impossible to administer any medicine without using force. Had the lady died in this state, and dissection been desired, a turgescence of the vessels of the brain, water effused into its ventricles, or some other deviation from the healthy state, would probably have appeared. Her death might then have been attributed to one or more of these circumstances.

"Viewing this case differently, and considering that she had been incessantly raving, till from exhaustion, she could rave no longer; that she had not closed her eyes for five successive days and nights; that weak broth had been the only sustenance allowed her, I inferred, that although there might be some disease in the brain, either congestion of blood, or effusion of serum, the patient was necessarily nearly worn out, and her life in danger.

"Under this impression, therefore, I immediately ordered her two glasses of old port wine, and two hours afterwards, three ounces of a decoction of bark with some of the tincture, as the only means of saving her life. In four hours, from my first seeing her, she was in a sound sleep, but only for a short time. Upon her awaking, the same quantity of decoction of bark was again given, when she slept three hours together. On the following morning, her life was comparatively safe; although she was still unconscious where she was, and took no notice of persons in the room; she no longer clenched her teeth or spit; but, when breakfast was offered to her, she put the cup naturally to her mouth; and after obtaining more sleep from a continuance of these remedies, she was able to answer questions correctly; in short, her irritability began to subside, and her sense of feeling to return, in some degree, from the moment they were first applied." 121.

Notwithstanding the fortunate issue of the above case, we should be loth to exhibit bark and port wine often, where the skin was parched—the eyes suffused with blood—the tongue brown, and the patient delirious.

In mental derangement, unaccompanied by delirium, the *irritability* may be very great, yet no immediate danger may be at hand. Here, also, our author recommends the stimulating plan. "Wine, bark, and musk, therefore, with the addition of henbane, hemlock, *tartar emetic, and fox glove* (curious auxiliaries to bark and wine !) may all, or each of them, be given."

Our author asks, is it not a mistake to suppose "that a quick pulse and a flushed countenance necessarily preclude the use of tonics and stimuli ?" To this question we would answer, "*yes, in general.*" And to Dr. Willis's next assertion that—these symptoms (quick pulse and flushed countenance) appear *more frequently* than otherwise to be the consequences of debility," we would answer—"No, in general." We would ask whether the symptoms above-mentioned, as "more frequently" appertaining to debility, be not two of the most usual concomitants on inflammation of all the great internal organs—on pneumonia, carditis, cephalitis, peritonitis, &c. ? How then can we lay down such a dangerous principle for the guidance of the young and inexperienced, as that these symptoms are to be generally looked upon as indicative of debility, and consequently forbidding every kind of depletion. Be it remembered that the above observations of Dr. Willis do not refer purely to insanity—but are general expressions—and he offers the following *very appropriate* illustration. "If a person, by shipwreck or other accident, be deprived of sustenance for many days together, shall we continue that deprivation because we find a quickened pulse and a flushed face follow the reception of very small quantities of food into his stomach ? " We wonder how Dr. W. could think of offering such an extravagant instance as an illustration. How many examples are there on record, where the application of heat after severe cold, and the introduction of food after long fasting, have quickly extinguished the remains of vitality in the part or whole of the system ?—Nothing can be more illustrative of the *caution* necessary in applying stimulation, than the example brought forward by Dr. Willis, as an exemplification of its utility. In extracting the following passage on practical points, we could not help remarking the *probabilities* so often introduced by our author.

"Although hemlock and henbane be valuable in allaying irritability, they are nevertheless dangerous, if administered without due caution; whilst one dose will moderately soothe, another of greater quantity will stupify. In some cases, irritation is even increased by very small doses, whilst the constitution is under the first and immediate influence of them.

"Tartar emetic, another valuable remedy, in irritable cases, requires also to be cautiously administered. Where a patient shall be in no danger, but incessantly talking, a quarter of a grain of tartar emetic, by nauseating, may restrain him; but if this medicine were injudiciously continued every hour for any space of time, he would *probably* become more irritable than ever, and weaker; while his voice and actions would deceive, and suggest the idea, that he was stronger, at the very time he might be approaching to a state of danger. The same result would, in all *probability*, follow the use of antimony in any form, of digitalis, or of the spiritus mindereri, if given too often, and in too large doses, without due attention to their effects.

"I purposely omit to mention opium, because, as it confines the bowels, and frequently produces watchfulness, I do not think it a desirable narcotic in this disorder." 126.

In concluding the subject of bleeding, Dr. Willis observes, "that it cannot be expected we shall ever be enabled to discover, by a view of the dead, however minute and close the inspection, *any thing whereby to assist us* in the cure of the disorders of the mind." Now, as Dr. Willis maintains, and we think justly so, that insanity results from *corporeal disease*; with what consistency does he maintain that *post mortem* researches can never assist us in acquiring a knowledge of that *corporeal* disease? Is this the case in other diseases of the material fabrick?—We maintain, then, that the prosecution of pathological investigations is as necessary in insanity as in any other disorder—and that although we have not yet been able to ascertain that "*peculiar state of the nervous system*," which Drs. Cullen and Willis conceive to be, *probably*, the proximate cause of insanity, we are not to be deterred from the pursuit by our hitherto want of success.

*The Mental Disorder.* After persisting in our efforts to restore the corporeal health perfectly, we must endeavour, says Dr. Willis, to restore the mind also. "Sound and refreshing sleep, he considers as the greatest desideratum, both in cure of the body and the mind." "We should, therefore, endeavour to procure a continuance of it for many nights together." Having by this means, in some measure, restored the tone of the nerves, it should be our next care to divert the mind of the patient from his morbid association of ideas, and direct his attention to other points. Narcotics are to be employed to procure sleep, and bodily exercise for the purpose of producing a pleasant degree of fatigue.

"As to the second (the diversion of the mind) we know that a man of sound mind, when absorbed in thought, or deeply engaged in business, is insensible to what is passing in his presence; we can hardly hope, then, by the introduction of friends, the exhibition of pictures, the representation of plays, or similar amusements, to make any useful impression upon one of unsound mind; but since we have seen the former capable of being roused by any thing that suddenly alarms his mind, or irritates his body, it is reasonable to infer that any shock which the latter shall receive, be it from what cause it may, will produce in a degree a similar effect, that may in his state prove of use to him. What then is likely to be better calculated for the purpose than an emetic? It not only cleanses the stomach of much viscid matter, and thus assists in restoring the general health, but by the nausea it creates, and the disturbance it occasions, rouses the patient, and dissipates his thoughts. Moreover, by its action upon the whole frame, it excites perspiration, produces fatigue, promotes sleep, thereby conducing to the restoration of the tone of the nervous system, and to a probable return of that state, which is requisite for the steady and healthy operation of the intellectual faculties." 136.

The testimonies of Monro, Cox, Hill, and others towards the utility of emetics, are brought forward by our author, and there is no doubt or question of their occasional efficacy in this disease, notwithstanding the contrary opinions of Halloran and Haslam. Blisters, at a distance from the head, are strongly recommended by Dr. Willis. After citing opinions for and against this practice, Dr. Willis quotes a passage from the favourite author of the late Dr. Gregory of Edinburgh—Gaubius.

"Gaubius has evinced, in his treatise on these complaints, a greater knowledge of the method of treatment in the cure of them than any other physician, antient or modern. His remarks apply so closely to this part of my subject, that I shall offer no apology for quoting him at some length. 'If experience teaches us,' says he, 'as I have shewn above, that the mind perceives differently according to the various conditions of the body to which it is joined, and that she may be disturbed by the body in her operations, and at some times be hindered from thinking as she would, and at other times be compelled to think according as the body commands; doubtless a physician making a proper use of the power which their intimate alliance affords him, can by his medicines so act on the body as to give relief to the mind, though he pay no regard at all to the causes or effects, which are in, or are feared to be in the body.

"'He hath such things at hand which his art furnisheth him with, that can compose the mind though ever so violently agitated, that can divert her from thinking upon what she is ever so closely bent upon, that can oblige her to keep holiday by erasing for a while all her ideas, and introducing a kind of general oblivion. An opiate, by laying the body fast asleep, can perform all these things.

"'The physician hath auxiliaries at his command, which may recall the mind again, when she hath ceased to think; or, at least, after she hath lost all consciousness of thought, which can interrupt her too attentive and close reflection; finally, which can dissipate ideas that have been too long present, and substitute others in their room. Such is the power and virtue of those remedies which stir the humours, irritate the nerves, or give pain'" 144.

After this, Dr. Willis introduces several judicious observations, some of which we shall notice. There are particular symptoms of recovery that are often misinterpreted by the inexperienced practitioner, such as pain in the head, and a general aching and soreness, complained of after remedies have been applied for some time. These are rather proofs of a return of natural feeling than of the impropriety of the remedies that have been used. Relapses are always to be expected—but, in favourable cases, an increase of amendment may be rationally expected after each relapse, till the cure is effected. Daily improvement, however, is not to be looked for. We must draw our conclusions from the occurrences of a week or a fortnight. "The pulse will not fall from 130 to 70, as rapidly as in other complaints:—it may continue about 100 for some weeks, without causing any alarm, provided the patient be supported. As irritation subsides, the pulse gradually diminishes." p. 149. If a patient, after being perpetually restless, can sit quiet in his chair for half an hour, we may judge favourably of him though his delusions be equally strong as before. When he remains composed for whole days together, proving thereby a great subsidence of nervous irritation, we may look for a return of reason. This takes place very gradually. As the bodily health improves, and the nerves recover their tone, the mind gains strength also. The patient begins to feel his situation, to reflect upon his delusions, to doubt as to their truth or fallacy;—and, after supposing that he may possibly be in error, he ultimately becomes so satisfied of the absurdity of his ideas, that he wonders how he could ever have believed them to be true. No man, Dr. W. thinks, can be considered as cured—that is, of sane mind, "until he freely and voluntarily confesses his delusions." This can only apply to mono-mania, or a delusion on a single topic. Many become insane, without any fixed point of hallucination, and who, on recovery, cannot tell what they may have said or done previously.

Upon the moral management of the insane, Dr. Willis makes several judicious though not very novel remarks. Separation from friends and home is the first step; and the next is, to bring the froward and untractable patient under discipline. This must be mild or rigid, according to the disposition and previous habits of the insane. Speaking of the *waistcoat*, Dr. W. very properly observes as follows:—

"Some think that soothing measures alone would answer the end; these, therefore, reprobate the use of the *waistcoat* as needlessly severe, which, if more experienced and better informed, I am confident, they would be induced to commend, as the kindest and most benevolent mode that can be adopted. Imagine, for instance, a person in a high state of delirium, running about almost naked, turning over every thing near him, threatening to kill any one who dares to touch him, at the same time loud and frightful in his voice, and so totally lost as to be quite insensible to any thing said to him. Can words alone soothe and soften in such a case? Can they have any effect, during his present state, in preventing him from either being starved to death in his nakedness, or from wearing himself out by his violence and want of rest? How shall quiet, so requisite and essential, be obtained for him in this state? Surely the only way is by restraining and subduing him. Without such control, too, how are we to administer his food and medicine, or how prevent him from injuring himself or others?" 162.

The straight waistcoat is not only required for the purposes above described—it is salutary in itself. It alarms the mind—leads to reflection—subdues the spirit—and produces rest. "In fact, says Dr. W. so great are the benefits arising from this mode of restraint, that it would argue more than weakness, and little less than inhumanity, not to enforce it when manifestly required." Dr. Willis illustrates the efficacy of a straight-waistcoat and tonic treatment by the following case.

"A gentleman, who had been out of health for some time, being rendered anxious by business, gradually lost his natural rest, and becoming incoherent in his language and conduct, I was requested to visit him. I found him in great agitation, incapable of sitting still, passing from room to room, holding conversations with persons whom he imagined to be present. Amongst other things, he fancied that a group of people were dancing under the grate, insects crawling upon his clothes, and animals playing upon the carpet. Improbable as he acknowledged this to be, still the more he looked, the more he was convinced of the fact. His pulse was 96, skin hot, face and eyes suffused with blood, his pupils dilated, and upper eyelids much elevated. I learnt that his bowels had been acted upon by medicine, and that he had been without sleep for three nights. A proper person having been procured to attend upon him, a draught composed of decoction of bark and camphor mixture was ordered to be given every three hours. He grew more irritable, passed a very unquiet night, and in the morning showed a disposition to be violent, threatening to jump out of the window, unless permitted to leave his room. The waistcoat being put on, he was confined in bed. His involuntary and uncontrollable efforts were thus checked, and he fell asleep for four hours; at the moment of awaking he was very clamorous, but, on continuing the medicine, went to sleep again, had a very good night, and awoke calm and collected, observing he had been under strange delusions. He was advised to remain in bed, to take two grains of calomel, and continue the medicine three times that day. On the following morning, having passed a second good night, his pulse was 68, skin cool, countenance natural, and his language and conduct so perfectly consistent, that, my attendance became unnecessary. He nevertheless, by his own desire, took the medicine for a few days longer, and remains quite well." 166.

We have extracted the above case as exhibiting a mode of practice almost the reverse of that generally pursued. Most of our modern doctors initiated in the mysteries of insanity, would have employed a very different plan in the above case—and we leave it to be settled by them, as to the eligibility of the tonic treatment here recommended. We attach the more importance to Dr. Willis's statements, as his opinions are pretty evidently founded on the experience of his grandfather, of well-known celebrity in this line.

Dr. W. remarks that the establishment of a cure will greatly depend upon the period that has elapsed between the commencement of the disorder and the application for advice.

"The prognosis of a patient's return to reason is the more favorable where bodily disease is most conspicuous. His life, however, may for this reason be in greater danger. Still, provided skilful means are early administered, and the patient survives, the recovery of the mind is almost as certain to follow that of the body, as night follows day. But if, by improper treatment in the first instance, the cure of the bodily disease is prevented from taking place within a reasonable time, a fixed state of insanity is often an inevitable consequence. Again, should the health of body improve, and the mind in the mean time evince no signs of amendment, the prognosis of its restoration becomes less favorable, and the case then approaches nearer to insanity, thereby lessening the hope of a cure." 168.

In this place Dr. Willis enters into a defence of his grandfather's evidence before the House of Commons, against some strictures put forth by Dr. Haslam. It appears that Dr. Willis had stated, "that of patients placed under his care within three months after the attack of the disease, nine out of ten had recovered." Upon this statement Dr. Haslam comments thus :—"He should have been more satisfied as to the *truth* of that assertion, had it been plausibly made out; but he required some other evidence than the *bare assertion of the man pretending to have performed such cures.*" *Haslam*, p. 251.

This is severe—indeed, illiberal language—and such as we unfortunately too often meet with in the discussions carried on between gentlemen who pursue *particular branches* of the profession. It would almost seem that, in many cases at least, liberality of sentiment is narrowed in proportion as a medical man confines himself to an exclusive branch of the healing art.

Dr. W. quotes a passage from the work of Mr. Hill, of Chester, reflecting on Dr. Haslam's criticism, and corroborating the statement of the elder Willis respecting the successful treatment. The younger Willis also avers that the passage from his grandfather's answers only applied to the *high* state of insanity, or "*delirium and derangement cum febre,*" under which the King then laboured.

*Low State.*—This, Dr. W. remarks, is characterised by an unusual lowness, sometimes amounting to despair, and frequently accompanied with the desire of suicide.

"The symptoms which precede derangement in the Low, are very different from those of the High state. In the place of hurry and unusual violence, we find great apathy, and profound listlessness. A patient in the former state, instead of enjoying the company of his friends, delighting in his accustomed amusements, and following his ordinary occupations, shuns society and seeks solitude; he prefers his bed to any other place; neglects his dress, disregards his food, and is averse to exercise: he is even unwilling to move from his seat; but if he does, it is with a slow step; he will sit for hours together without seeming to be capable of motion. He seldom speaks; and if spoken to, he does not answer till the question has been put to him two or three times; he is, in short, totally lost and regardless of every thing, except the subject upon which his mind appears to be wholly employed.

"A pallid and fixed countenance, dull eye, scantiness of secretions, loss of appetite, languid circulation, long and deep inspiration or sighing, all accompany this inactivity of mind. The patient now obtains very little sleep, yet, owing to the general torpidity of his constitution, he is with difficulty roused. Disturbed and restless nights succeed, more or less attended with fever and debility. After a continuance of this state, perpetual watchings, confusion of ideas, and derangement, may ensue without any other apparent symptoms. Very often, however, indigestion is the primary affection." 185.

Unless the preceding symptoms are attended to, the fever increases—the patient's countenance betrays alarm and anxiety—he grows suspicious of his dearest friends—is surrounded with imaginary horrors, and dreads to be left a moment alone. At length the delusion becomes fixed—his mind is impressed with some false idea—his life appears irksome—and death desirable.

The same remote causes which occasion the high state, may also occasion this; "but why such different states should arise from similar excitements, it may be difficult to determine."

"The remote causes may, as in the high state, be considered under two heads, according as they primarily affect the body or the mind.

"Under the first division, we may include whatever by immediate application offends or injures the stomach and other abdominal viscera, creating indigestion, the effects of which are strongly exemplified in the subsequent case.

"A young lady, after eating some heavy paste, had been attacked with a sensation of burning heat at the pit of the stomach, which increased till the whole of the upper part of her body, both externally and internally, appeared to her to be in flames. She rose up suddenly, left the dinner table, and ran out into the street, where she was immediately followed, and brought back. She soon came to herself, and thus described her horrible ideas, that she had been very wicked, and was dragged into the flames of hell. She continued in a precarious situation for some time. Whenever she experienced the burning sensation, of which she first complained, the same dreadful thoughts recurred to her mind. She seized hold of whatever was nearest, to prevent her from being forced away, and such was her alarm, that she dreaded to be alone. This lady had been long distressed by family concerns, and harassed by restless and disturbed nights, which had greatly injured her health.

"Persons addicted to drinking spirits, especially in hot climates, frequently become dyspeptic, and are sometimes the subjects of the Low state, which may likewise be the consequence of hypochondriasis and hysteria.

"Under the second head of exciting causes, we may consider whatever operates so powerfully upon the mind, as to paralyse, as it were, the whole frame, and in particular the viscera of the abdomen. Amongst such may be mentioned great anxiety of mind, severe disappointment, loss of reputation and property, long continued attention to any important concern, and accidentally killing a near relation or friend." 195.

A case of sudden delirium, terminating in suicide, is brought forward by our author from his favourite sepulchretum—the works of Shakspeare.

Many of the remedies recommended in the high state are proper in the low. Dr. Willis considers sleep as the first thing to be procured—and next a proper state of the digestive organs. We must be careful to distinguish the restlessness which is a consequence of nervous irritation, from that which is an effect of disordered bowels. In the latter case, bark would increase the malady, by lying heavy in the first passages. Emetics and purgatives are therefore proper at the beginning, "which should be followed by medicines that tend to restore the tone of the stomach and other abdominal viscera, such as mild alteratives, tonics and cordials."

The prognosis in this state is less favourable than in the high, because the attack is commonly more insidious, and much time is suffered to elapse before advice is sought. "It is remarkable," says Dr. Willis, "that patients who fancy they are poisoned or suffering internally, are less likely to recover than those who imagine themselves ruined in fortune, or disgraced by crimes." Dr Willis traces this low state to the *lethargus* of the ancients, as he does the high state to the *phrenitis* of the same people. We do not think it is worth while to enter into this learned dissertation, which leads to no practical purpose in the end.

Between these two extremes, the high and the low state, there are various intermediate shades, which cannot be described, and which must be left to the discrimination of the practitioner. We shall conclude with the following very judicious piece of advice from the pen of the celebrated Monro.

"'The only cautions necessary for those unacquainted with this 'complaint, are, not to be too hasty in their evacuations, nor to carry 'them beyond the strength and constitution of the patient; never to 'lose a proper authority over him, which they will always find neces'sary for his management; not to be imposed upon by his cunning 'artifices; nor to give him up too soon as an incurable. As to medi'cines, there can be no particular directions; all that are proper in 'other distempers will be found of use in this when applied with judg'ment." 210.

We have thus presented the leading features, facts, and opinions, contained in Dr. Willis's lecture. And although we cannot go so far as our author in recommendation of the tonic treatment in mania, yet we have seen enough to convince us that a very common error is committed in amalgamating the excitement of mania with the delirium of inflamed brain, and treating it with active depletion on that principle. Practitioners will find, sometimes when it is too late, that maniacal patients, however violent their symptoms, will not bear depletion like patients labouring under inflammation of the brain or its membranes.

